# Research reviews on myosin head interactions with F-actin

**DOI:** 10.1186/s42649-024-00099-8

**Published:** 2024-08-28

**Authors:** Yoon Ho Park, Gang San Song, Hyun Suk Jung

**Affiliations:** Department of Biochemistry, College of Natural Sciences, Kangwon National University, Chuncheon, 24341 Republic of Korea

**Keywords:** Myosin II, Two-headed binding, Regulatory light chain, cryo-EM, Cross-bridge cycle

## Abstract

The sliding filament theory and the cross-bridge model have been fundamental in understanding muscle contraction. While the cross-bridge model explains the interaction between a single myosin head and actin filament, the native myosin molecule consists of two heads. This review explores the possibility and mechanism of two-headed binding in myosin II to the actin. Recent studies using electron tomography and resonance energy transfer have provided evidence in support of the occurrence of two-headed binding. The flexibility of the regulatory light chain (RLC) appears to play a significant role in enabling this binding mode. However, high-resolution structures of the RLCs in the two-headed bound state have not yet been reported. Resolving these structures, possibly through sub-tomogram averaging or single-particle analysis, would provide definitive proof of the conformational flexibility of RLCs and their role in facilitating two-headed binding. Further investigations are also required to address questions such as the predominance of two-headed versus single-headed binding and the influence of the state of each of the heads on the other. An understanding of the mechanism of two-headed binding is crucial for developing a comprehensive model of the cross-bridge cycle of the native myosin molecule.

## Introduction

The sliding filament theory of muscle contraction, a fundamental concept in the field of muscle physiology, describes the mechanism by which muscle fibers generate force and movement (Huxley 1957, 2004; Andersen 2004). This theory proposes that muscle contraction occurs as a result of the interaction between two proteins, actin and myosin, which are found within the muscle fibers. According to the theory, during muscle contraction, the sliding action of the myosin filaments along the actin filaments shortens the muscle fiber to generate force. Huxley conducted additional research using electron microscopy to confirm the overlapping nature of the actin and myosin filaments (Huxley 1963). This study provided evidence for the existence of actin-myosin linkage, as well as the sliding filament mechanism proposed by the theory. These findings supported the sliding filament theory and contributed to the current understanding of the molecular basis of muscle function. Based on the collective evidence (Hanson and Lowy 1959; Haselgrove and Huxley 1973; Begg et al. 1978), the sliding filament theory underwent further development and refinement from which the cross-bridge model emerged. This model proposes that the interaction between actin and myosin occurs through a series of temporary attachments, or “cross-bridges,” which form and break as the myosin heads bind to and become detached from the actin filaments (Spudich 2001; Zeng et al. 2004; Galler et al. 2005). The cross-bridge model builds upon the original sliding filament theory and provides a more detailed understanding of the molecular mechanisms underlying muscle contraction.

This review aims to address the ongoing debate between single-headed and two-headed myosin binding mechanisms in muscle contraction by proposing new directions for future research. We emphasize the critical need for high-resolution structural studies of the heavy meromyosin (HMM) molecule, which contains both myosin heads, interacting with actin filaments. Specifically, we highlight the importance of resolving the high-resolution structure of the regulatory light chains (RLCs) in the acto-HMM complex. Such structural information is crucial for definitively determining whether myosin II engages in single-headed or two-headed binding to actin filaments. By synthesizing recent experimental evidence and identifying key areas for investigation, we seek to identify critical areas of focus for future studies that could resolve this longstanding question and advance our understanding of the molecular mechanisms underlying muscle contraction.

## Structural features of myosin II

**Fig. 1:**
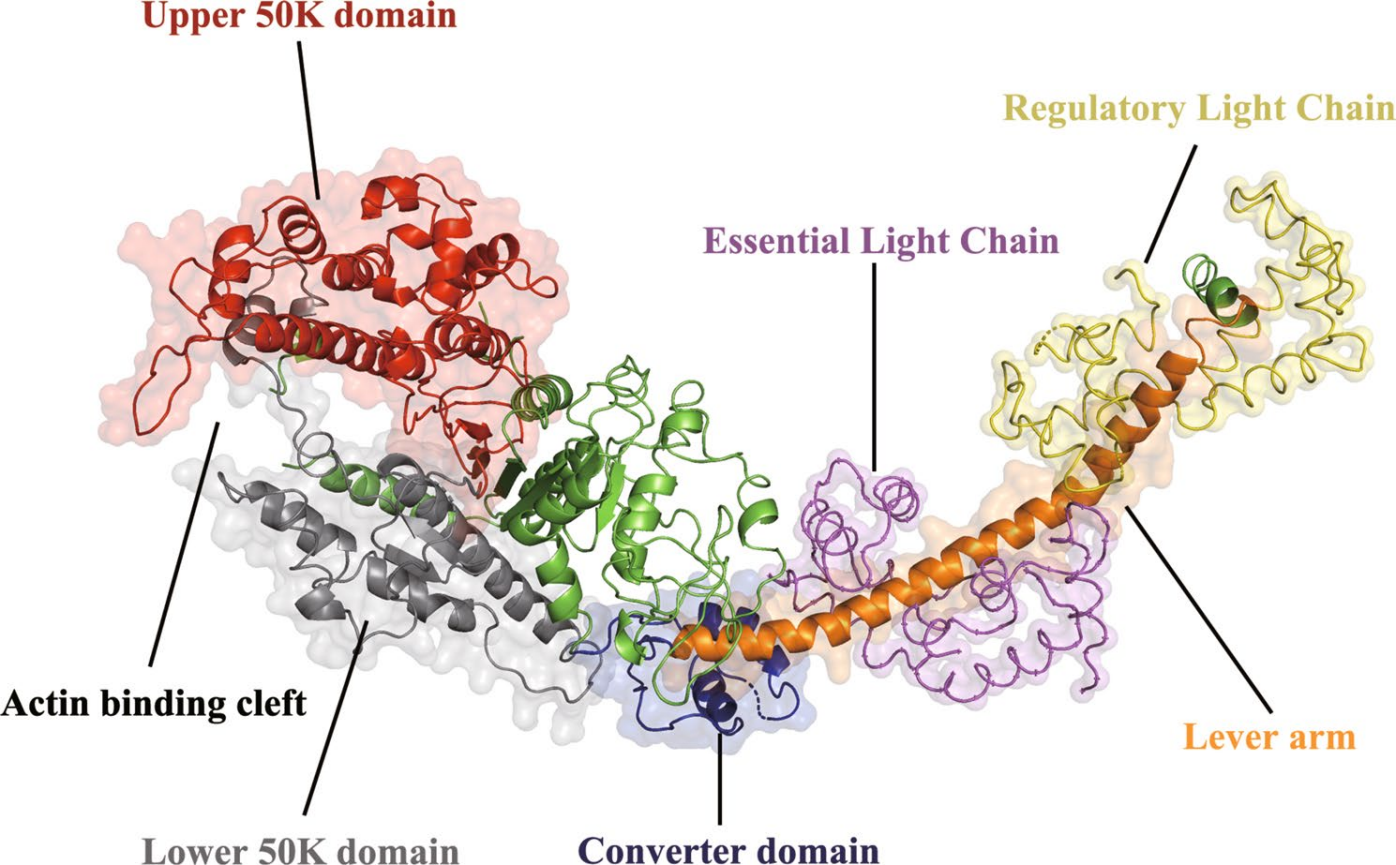
Composition of the myosin head domains

To understand the concept of a cross-bridge, it is necessary to first comprehend the structure of myosin. Myosin molecules were first visualized by electron microscopy (RV 1961), which showed the myosin II molecule to have a globular region at one end of the molecule attached to a long, fibrous tail (ELLIOT and OFFER, 1978). Each myosin monomer is composed of six polypeptide chains, among which are two heavy chains that each have a molecular weight of ∼ 220 kDa. These heavy chains each contain 2 IQ motifs in the neck region which are bound to an essential light chain (ELC) and a regulatory light chain (RLC) which are both ∼ 20 kDa. The C-terminal halves of the two heavy chains dimerize to form the highly elongated coiled-coil alpha-helical tail. Structurally, the myosin head consists of a few distinct and important domains (Fig. 1): the lower 50 K, upper 50 K, converter, and lever arm domains. The 50 K domains are involved in binding to actin. Among the 50 K domains, the lower 50 K domain appears to be the primary actin-binding site and an actin-binding cleft exists between the upper and lower 50 K domains. The converter domain is joined to the lever arm domain and communicates structural changes that enable the lever arm domain to generate force via the ATPase activity of myosin.

The myosin structure was obtained from the Protein Data Bank in Europe (PDB ID: 2MYS), and PyMOL software was used to visualize the surface and structure. Each domain is colored differently and is presented in the same unique color.

The cross-bridge cycle, the fundamental mechanism of muscle contraction, is used to describe the interaction between the myosin heads and actin filaments. The cycle begins with the myosin head in a high-energy configuration, strongly bound to ATP. As the myosin head binds to actin, the ATP is hydrolyzed into ADP and inorganic phosphate (Pi), which causes a conformational change in the myosin head. This change is primarily due to the movement of the converter domain, which leads to the rotation of the lever arm domain to produce the power stroke. During the power stroke, the pulling action of the myosin head on the actin filament generates force and causes the sarcomere to shorten. After the power stroke, ADP is released, and a new ATP molecule binds to the myosin head, causing it to detach from actin. The myosin head then undergoes a recovery stroke to return to its original high-energy configuration, ready to begin the cycle again.

## Single-headed interaction with F-actin

The mechanism of muscle contraction is comprehensively explicated by the sliding filament theory and the cross-bridge cycle. The latter effectively elucidates the binding cycle of a single head of myosin to actin. Nonetheless, this phenomenon has not yet been fully explained. Notably, one such poorly understood aspect is the manner in which two myosin heads are mobilized. Myosin V, a specific type of myosin, was found to engage in movement that resembles walking (Walker et al. 2000; Moore et al. 2001; Molloy and Veigel 2003) and that the elongated neck of myosin V accounts for this phenomenon (Sakamoto et al. 2003). In contrast, the shorter neck of myosin II, compared to that of myosin V (Rayment, et al., 1993b), directly affects the step size of the former, which is operationally defined as the amount of filament sliding that one myosin head produces during one ATPase cycle (Burton 1992). The measured step size of a single head of myosin II is 4 ∼ 5.3 nm (Molloy et al. 1995; Kitamura et al. 1999), although certain steps are longer at 11 ∼ 30 nm (Kitamura et al. 1999). This variety in the myosin step size has given rise to uncertainty regarding the movement of myosin heads upon attachment or detachment from F-actin. Considering that the heads could possibly move by undertaking swinging, walking, or jumping motion, various models have been proposed, although consensus has yet to be reached (Yanagida and Iwane 2000; Spudich 2001; Wendt et al. 2001; Kovacs et al. 2007). A comprehensive understanding would necessitate an investigation as to whether the two heads of myosin II could simultaneously bind to F-actin in the same state.

The current understanding of the interacting structure between a myosin head and actin is well established (Schroder et al. 1993; Banerjee et al. 2017; Fujii and Namba 2017). Additionally, the binding state of each nucleotide of myosin S1 has been comprehensively clarified (Houdusse et al. 2000). Consequently, the interaction cycle of a single head of myosin has been thoroughly explained (Banerjee et al. 2017; Mijailovich et al. 2017). However, two-headed binding in the rigorous structural state could be problematic. The lever arm of binding myosin S1 is oriented parallel to the direction of the short lever arm, to enable connection with the other binding myosin S1 without causing any collisions with the light chains. Despite the occurrence of conformational changes (Tama et al. 2005; Pylypenko and Houdusse 2011), collisions with the RLC were shown to still persist. Additionally, the binding of the first myosin head lowers the affinity of the second myosin head towards the adjacent actin monomer (Reshetnyak et al. 2012), with the result that the myosin proceeds with single-headed binding.

## Two-headed interaction with F-actin

Contrary to the conventional approach in which myosin binds via a single head, the proposed two-headed binding presents structural issues. One such problem concerns the structure of actomyosin, which binds with the myosin structure via sub-fragment S1 (acto-S1). (Rayment et al. 1993a; Schroder et al. 1993; Lorenz and Holmes 2010). The acto-S1 model indicates that further binding of myosin S1 is not feasible without causing distortion. In accordance with this model, the great distance between the myosin rod domains simply prevents connection without uncoiling myosin sub-fragment 2 (S2), or without causing distortions within myosin S1 (Chakrabarty et al. 2002). Resolving the structure of actomyosin between the myosin head and F-actin, similar to the study of myosin S1, could be used to demonstrate the way in which myosin binds to F-actin via two-headed interaction (Rayment et al. 1993a; Schroder et al. 1993; Lorenz and Holmes 2010). However, as myosin S1 has a structural limitation in that it is composed of only a single head, it cannot confirm the occurrence of two-headed binding. Therefore, to ensure that the structure more closely approximates the native structure, the heavy meromyosin (Hojjatian et al. 2021) has been used.

Resonance energy transfer investigations related to the RLC of myosin have indicated the possibility of two-headed binding. This phenomenon is thought to be facilitated by the uncoiling of the S2 domain of HMM, which ensures that the two head-rod junctions remain in close proximity while the myosin head is simultaneously distorted (Chakrabarty et al. 2002). In addition to this, other researchers reported that the strong binding conformation of the myosin molecule in the state of structural rigor involves the presence of parallel light chains on both heads, which implies that the two heads of myosin are closely aligned during actin binding (Lidke and Thomas 2002).

Moreover, investigations based on structural visualization with the aid of tomography have revealed the occurrence of two-headed binding. An image captured by transmission electron microscopy (Mohanty and Mohanty 2023) of a slender section of fibers from insect flight muscle demonstrates electron density that indicates the binding of two myosin heads to F-actin in a state of rigor (Chen et al. 2002; Liu et al. 2006; Wu et al. 2010). Additionally, the atomic models adjusted to the density suggest a more complex structural interaction between the two heads.

Recently, a new study in which electron tomography was utilized to examine the skeletal sarcomere structure of mice was reported. This study developed models to illustrate two-headed binding to a thin filament and the binding of two heads to two different actin filaments (Wang et al. 2021, 2022). In fact, two-headed binding has been reported to occur not only in skeletal muscle myosin but also in smooth muscle myosin (Hojjatian et al. 2021). However, the high-resolution structure of RLCs has not yet been confirmed.

The reason for this is the flexibility of RLC, which, ironically, plays a significant role in enabling both single-headed and two-headed binding. This flexibility of RLCs is crucial for allowing necessary conformational changes in myosin heads during the binding process. In single-headed binding, RLC flexibility enables optimal positioning of the myosin head relative to actin, potentially enhancing the efficiency of the power stroke (Guhathakurta et al. 2015). For two-headed binding, this flexibility becomes even more critical, potentially facilitating the simultaneous engagement of both heads with actin filaments, overcoming structural constraints that would otherwise prevent such binding. The dynamic nature of RLCs may contribute to the fine-tuning of force generation and transmission in muscle contraction in both binding modes. However, this very flexibility presents challenges in obtaining high-resolution structural data. To fully understand these mechanisms, high-resolution structural studies of the RLCs bound to myosin heads in both single- and two-headed binding states are essential. Such structural data would not only provide definitive proof of the conformational flexibility of RLCs but also elucidate how this flexibility enables and regulates different binding modes. This structural information is crucial for developing a more detailed model of myosin-actin interactions. Such a model could potentially resolve the ongoing debate regarding the prevalence and functional significance of single-headed versus two-headed binding in muscle contraction. Furthermore, it would provide insights into the molecular mechanisms underlying the cross-bridge cycle of the native myosin molecule.

Understanding the implications of RLC flexibility and the potential for two-headed binding could significantly impact our overall understanding of the cross-bridge cycle. If two-headed binding is confirmed, the current model would need to evolve from describing a single myosin head interaction to a more complex representation of coordinated dual-head action. A detailed structural analysis of the flexible RLC could reveal specific stages based on the binding positions of each head, providing insights into the molecular basis of the power stroke and force modulation. This refined model could bridge the gap between single-molecule studies and whole muscle fiber behavior, potentially explaining variations in step size and force production under different conditions. Ultimately, incorporating two-headed binding and RLC flexibility could lead to a more dynamic and physiologically relevant model of muscle contraction, better reflecting the complexity of in vivo muscle function.

Table 1 Summarizes the key papers that support the single-head and two-head binding models of myosin II. Although the conventional approach has been to consider myosin as binding via a single head, with several studies providing evidence to support this view, recent investigations utilizing advanced techniques such as resonance energy transfer and electron tomography have yielded results that indicate the possibility of two-head binding. This ongoing debate underscores the need for further research to elucidate the native structure and binding mechanism of myosin II.

**Table 1:**
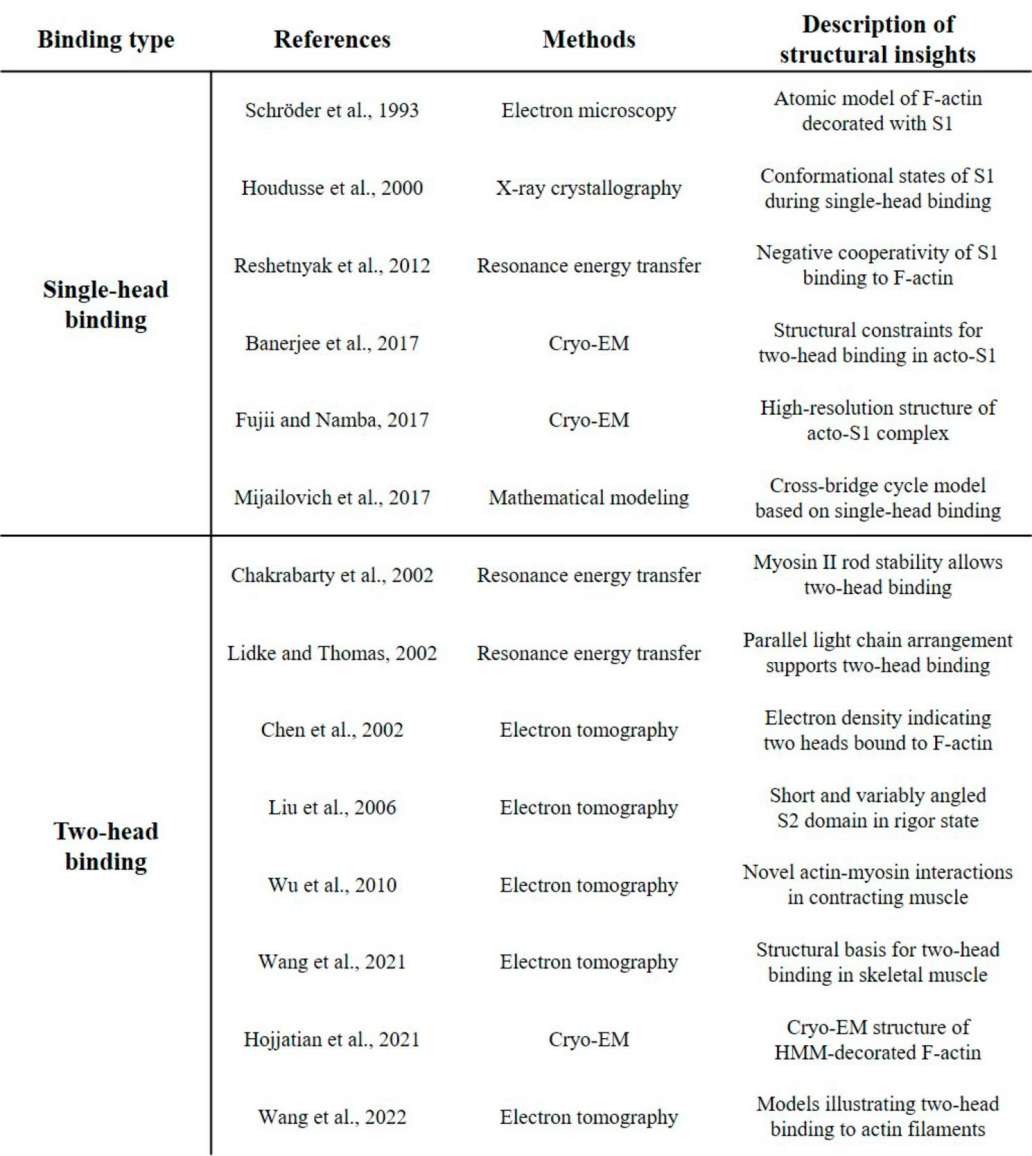
Key papers supporting single-head and two-head binding models of myosin II

## Conclusions

The scientific concept of muscle contraction has been extensively studied. The sliding filaments theory elegantly explains the process whereby muscle fiber shortens through the interaction of thick and thin filaments. The cross-bridge cycle, which describes the intricate structure of this interaction, focuses primarily on one head of the myosin molecule despite its native structure consisting of two heads. To develop a more comprehensive model, it would be necessary to identify whether both heads can simultaneously bind to F-actin. In order to arrive at a definitive conclusion on the phenomenon of two-headed binding, it was necessary to conduct an experiment involving a two-headed structure modeled on HMM. Unravelling the high-resolution structure of large protein complexes would necessarily require cryo-EM studies. However, the binding of HMM to F-actin has not been structurally resolved at present. The tomography technique was used to propose the density of HMM bound to F-actin via both of the two HMM heads simultaneously. The limitation of the electron density in explaining the bending of part of the RLC component necessitates the use of atomic resolution. Recently, the sub-tomogram averaging method has enhanced the resolution to a high level, and single-particle analysis can also determine the binding of HMM. These advancements are able to confirm the way in which native myosin heads bind and provide insight into the cross-bridge model, not only for the single-head cycle but also for the two-head cycle mechanism, that is, the cross-bridge cycle of the native myosin molecule. Further investigation is required to address accompanying questions, such as the preponderance of binding formation and the influence of the state of each head on the other. In cases of single-headed binding, the location of the remaining myosin head and the mechanism that regulates the cross-bridge cycling process with both heads in sequence necessitate thorough exploration and comprehension. These domains demand meticulous inspection and comprehension.

## Data Availability

The data presented in Figure 1 and Table 1 were generated by the authors for this review article. Figure 1 was created using the PyMOL software and the myosin structure from the Protein Data Bank (PDB ID: 2MYS). Table 1 was created by the authors to summarize the key findings from the cited literature. All other data discussed in this review article are from previously published studies and are cited accordingly. No new datasets were generated or analyzed during the writing of this review.

## Abbreviations

RLCs: Regulatory light chains
RLC: Regulatory light chain
ELC: Essential light chain
ATP: Adenosine triphosphate
ADP: Adenosine diphosphate
ATPase: Adenosine triphosphatase
PDB: Protein data bank
Pi: Phosphate
F-actin: Filamentous actin
HMM: Heavy meromyosin
TEM: Transmission Electron Microscope
Cryo-EM: Cryo-electron microscopy
